# First referral to an integrated onco-palliative care program: a retrospective analysis of its timing

**DOI:** 10.1186/s12904-020-0539-x

**Published:** 2020-03-12

**Authors:** Claire Barth, Isabelle Colombet, Vincent Montheil, Olivier Huillard, Pascaline Boudou-Rouquette, Camille Tlemsani, Jérôme Alexandre, François Goldwasser, Pascale Vinant

**Affiliations:** 1 Unité Mobile de Soins Palliatifs, Hôpital Cochin, AP-HP Centre, Paris, France; 2 Université de Paris, Public Health, Paris, France; 3 Oncologie médicale, Hôpital Cochin, AP-HP Centre, Paris, France

**Keywords:** Integration of oncology and palliative care, Palliative care, Advanced Cancer, Shared decision making, End-of-life care

## Abstract

**Background:**

Palliative care (PC) referral is recommended early in the course of advanced cancer. This study aims to describe, in an integrated onco-palliative care program (IOPC), patient’s profile when first referred to this program, timing of this referral and its impact on the trajectory of care at end-of-life.

**Methods:**

The IOPC combined the weekly onco-palliative meeting (OPM) dedicated to patients with incurable cancer, and/or the clinical evaluation by the PC team. Oncologists can refer to the multidisciplinary board of the OPM the patients for whom goals and organization of care need to be discussed. We analyzed all patients first referred at OPM in 2011–2013. We defined the index of precocity (IP), as the ratio of the time from first referral to death by the time from diagnosis of incurability to death, ranging from 0 (late referral) to 1 (early referral).

**Results:**

Of the 416 patients included, 57% presented with lung, urothelial cancers, or sarcoma. At first referral to IOPC, 76% were receiving antitumoral treatment, 63% were outpatients, 56% had a performance status ≤2 and 46% had a serum albumin level > 35 g/l. The median [1st-3rd quartile] IP was 0.39 [0.16–0.72], ranging between 0.53 [0.20–0.79] (earliest referral, i.e. close to diagnosis of incurability, for lung cancer) to 0.16 [0.07–0.56] (latest referral, i.e. close to death relatively to length of metastatic disease, for prostate cancer). Among 367 decedents, 42 (13%) received antitumoral treatment within 14 days before death, and 157 (43%) died in PC units.

**Conclusions:**

The IOPC is an effective organization to enable early integration of PC and decrease aggressiveness of care near the end-of life. The IP is a useful tool to model the timing of referral to IOPC, while taking into account each cancer types and therapeutic advances.

## Background

The access to palliative care in oncology, for patients with advanced cancers, hinges on hospital-based palliative care consultation teams (PCT). Despite being recommended early in the course of advanced cancer [1–3] palliative care is often proposed late and after the stop of antitumoral treatments [4–6] with low impact on patient’s quality of life [7]. The reasons for this delay are now well known [4, 8–10]., Patients and family-related barriers for early referral often include negative image of palliative care and unwillingness to end anti-cancer treatments. Barriers related to medical staff often include belated discontinuation of anti-cancer treatment, insufficient awareness of palliative care, inaccurate prognosis assessment and inadequate communication skills to discuss bad prognosis. The organization of care also participate in this delay, since the intervention of PCT is usually based on the presence of uncontrolled psycho-physical symptoms or specific situations raising ethical questions, such as death requests.

Other modalities of PCT intervention have been developed in the last decade to improve integration of palliative care. Hui et al., established a consensual set of precisely defined referral criteria, distinguishing time-based criteria and need-based criteria [11]. Numerous studies evaluated the feasibility and efficacy of integrating palliative care at a specific time in the disease evolution. The systematic integration of palliative care in oncology at the diagnosis of advanced cancer has been shown first to be feasible without worsening patients’ anxiety or depression, in the specific population of non-small cell cancer patients [12]. In following studies, early and systematic palliative care intervention showed increasing quality of care for patients with advanced cancer, by improving quality of life, psychophysical symptom management, and decreasing aggressive care at the end-of-life as well as health costs [13–17]., These findings have been synthesized by a Cochrane meta-analysis [18]. Based on this literature, the American Society of Clinical Oncology now recommends that all patients with advanced cancer “receive dedicated palliative care services, early in the course of disease, concurrent with active treatment” [19].

However, with the increasing therapeutic progresses made in oncology [20], the duration of advanced phase of oncologic diseases increases. Palliative care resources being limited and variable across countries [21, 22],, patients should be referred to PCT at the right time and for the good reasons. These remain to be defined pragmatically, taking into account assessment of patients’ needs and local resources of care [23, 24].,

In our institution, the PCT has developed since 2005 a specific organization with the oncology ward. This integrated oncology and palliative care (IOPC) program can be described by some indicators of integration close to those collated by Hui et al [25], i.e. communication, cooperation and coordination between PCT and oncology services, specified timing of PCT involvement, referral criteria for PCT. Our program also includes a shared-decision making process, which can be retained as an additional indicator [26]. Indeed, as Maltoni et al pointed it [17], the active participation of PCT in the shared decision making process is essential to have an impact on indicators of aggressiveness of care in end-of-life. In a previous study, we found that the IOPC decreased the odds of receiving chemotherapy in the last 14 days of life and dying in an acute care setting [27]. The annual follow-up of these indicators shows that this impact is persistent over time [28]. The objectives of our study were to describe the patients’ profiles at the time of the first referral to IOPC program; the timing of this referral regarding the course of the disease; and the consequences of this program on patients’ trajectory of care at the end-of-life.

## Methods

### Setting

Cochin Hospital is a tertiary care hospital treating around 4500 new cancer patients each year, with an oncology ward and three other medical specialty wards (gastroenterology, pneumology, dermatology) that have an oncologic activity of care.

At the time of the study, the oncology ward consists in a 9-beds inpatients unit and an 11-beds outpatient clinics. Medical staff is made up of three attending physicians and three fellow physicians, all advising two residents for inpatients and three residents for the outpatient clinic ambulatory patients.

The PCT consists in 2.5 full time equivalent physicians, all being palliative care specialists, 2.5 full time equivalent nurses and one secretary assistant.

Social workers and psychologists collaborate with both teams.

### Organization of integrated palliative care in the oncology ward

The IOPC program has been developed as a specific organization involving the PCT and the oncology staff.

This organization relies on weekly multidisciplinary onco-palliative meetings (OPM), which are attended by both the PCT and the oncology staff, i.e. physicians, head nurses, social workers and psychologists. Physicians of the PCT are in charge of moderating, keeping record of each meeting and reporting any decision and its rationale in the patient’s health record. The oncologists choose to refer patients to these meetings regarding the following criteria: situation of incurability and necessity to discuss goals and organization of care to anticipate the trajectory of care. Discussions take into account expected benefit of treatment on survival and quality of life, proportionality of care, and patient’s preferences. Decisions may be to pursue or change antitumoral therapies, associated or not with the introduction of the PCT, or to provide palliative care only. These decisions are then submitted and discussed with the patient. Later on, patients are followed-up by both the referent oncologist and the PCT, if deemed appropriate, in consultations, outpatient clinics, or inpatient acute care setting. For all patients discussed, goals and organization of care can be updated at following OPM, up to patient’s death. A part of OPM is also dedicated to deceased patients to review the trajectory of care, and the aggressiveness of care near the end-of-life.

Along with this organization, inpatients or outpatients can be referred to the PCT in an on-demand way, before being discussed at the OPM, if they are presenting with urgent needs (urgent psycho-physical symptoms, urgent need for shared-decision such as serious complications that require discussion on the appropriate intensity of care).

**Fig. 1:**
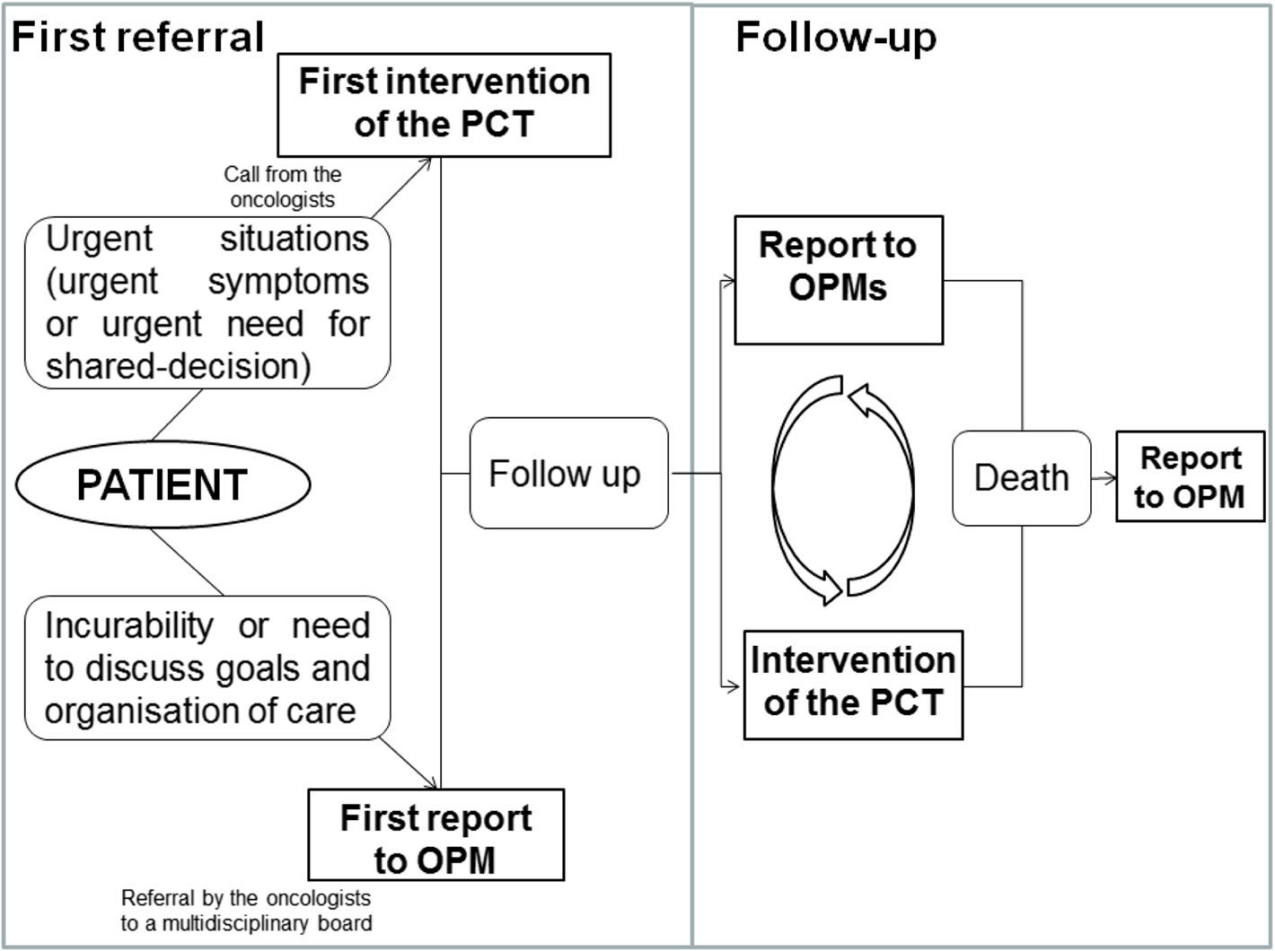
Organization of the integrated oncology and palliative care program (IOPC)

The first referral to IOPC is defined either as the first report at OPM or as the first referral to the PCT (Fig. 1).

### Study population and data collection

This study included the historical cohort of patients first reported at OPM between January 1st, 2011 and December 31, 2013. Non-eligibility criteria were: unavailability of patient’s health records, patients presenting with non-oncologic disease or curative cancer and first referral to OPM after death. All included patients were then followed-up until death or until December 31, 2016.

We collected data from patients’ files concerning: 1) social and clinical characteristics of patients: age and gender, primary cancer site, date of initial diagnosis, date at which disease was deemed incurable (i.e. without curative treatment options due to metastatic stage or inoperability, or both), indicators of social vulnerability (precarious living conditions, living alone, in charge of some relative, spouse diagnosed with serious disease, incapability to express wills from somatic causes), other health risks (active addictions, co-morbidities); 2) the context of the first referral to the IOPC program, regarding the course of disease and project of care: dates of first discussion at the OPM, first referral to the PCT and death, oncologic prognosis factors measured within 7 days of first referral to the IOPC program (ECOG [29] performance status (ECOG-PS), serum albumin level, serum C-reactive protein (CRP) level, serum lymphocyte count and serum Lactate Dehydrogenase (LDH) level), elements relative to the course of the disease (at diagnosis, tumour stability or positive response to last treatment, tumour progression), to the project of care (oncologic treatment to come, on course, definitely discontinued or not considered as future option) and to the setting of care (inpatient care, outpatient/ambulatory care); 3) indicators of end-of-life care for decedents: the number of new lines of antitumoral treatment received and length of survival after the first referral to the IOPC program, the place of death, whether the patient had been admitted to a palliative care unit 3 days or less before death, and whether the patient had received antitumoral treatment 14 days before death.

Part of collected data required physician’s expertise (i.e. diagnosis of incurability status) and a good knowledge of patient’s records to be found, most of variables being objective results (i.e. ECOG-PS or lab tests). We therefore had data collected by MDs experienced in both PCT and oncology teams practice (VM), monitored by senior MD researcher and statistician (IC), without actual blind double coding process.

### Statistical analysis

Data were analyzed by description of frequencies (percentage), means (±standard deviations) or medians (interquartile range) as relevant according to the normality of variable distribution and excluding patients with missing data.

In order to investigate the timing of the first referral to the IOPC program, taking into account the pace of progress of the disease, we defined the Index of Precocity (IP), computed for decedents only, as the ratio of the length of survival after first referral to the IOPC program by the length of survival after diagnosis of incurability. Its values lie therefore between 0 (referral to the IOPC program occurs late, close before death) and 1 (referral to the IOPC program occurs early after the diagnosis of incurability). As an example, the IP for a patient with a cancer diagnosed on January 2011 at a metastatic state, referred to the IOPC on March 2011 and deceased on June 2012 is: 15 months / 17 months = 0.88. For a patient with a cancer in metastatic evolution in September 2011, referred to the IOPC on September 2012 and deceased on December 2012, the IP is: 3 months / 15 months = 0.2.

$$ \mathrm{Index}\ \mathrm{of}\ \mathrm{Precocity}=\frac{\mathrm{Survival}\ \mathrm{after}\ \mathrm{first}\ \mathrm{referral}\ \mathrm{to}\ \mathrm{the}\ \mathrm{IOPC}\ \mathrm{program}}{\mathrm{Survival}\ \mathrm{after}\ \mathrm{diagnosis}\ \mathrm{of}\ \mathrm{incurability}} $$

## Results

### Patients’ social and clinical characteristics

**Fig. 2:**
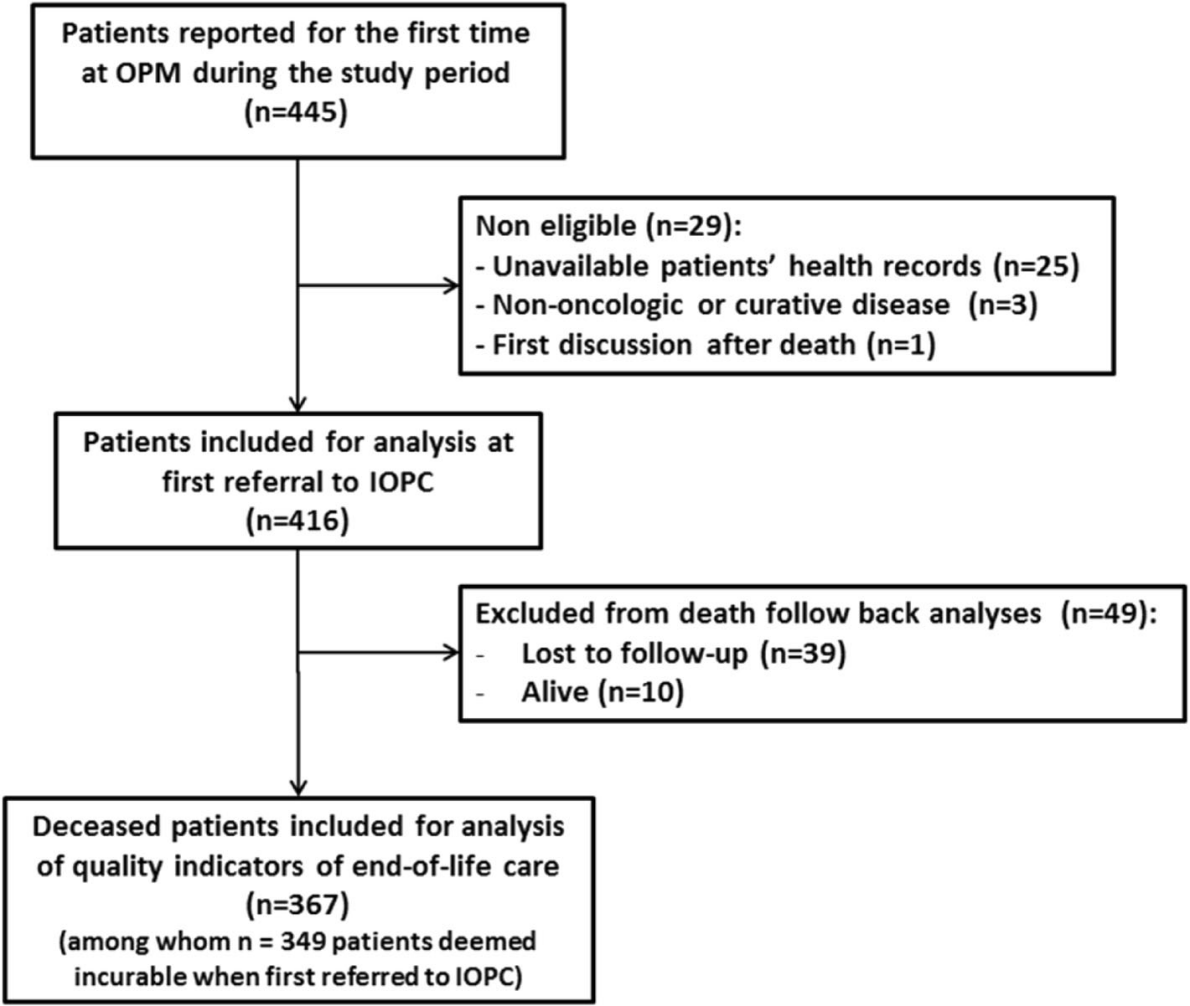
Flow chart

From January 1st, 2011 to December 31, 2013, 445 patients were reported for the first time at OPM. Among them, 416 patients were included for analysis (Fig. 2). Patient’s characteristics at the time of the first referral to the IOPC program are summarized in Table 1.

**Table 1:** Social and clinical characteristics of patients *(n = 416 patients)*

(*total number of subjects specified in the case of missing data)	N	(%)
**Age at first OPM (years),*mean (SD)***	*62.0*	*(15.2)*
**Male**	249	(59.9)
**Psychosocial and health factors of vulnerability**		
Precarious living conditions, (*n = 410)	57	(13.9)
Living alone (*n = 408)	118	(28.9)
In charge of some relative (*n = 410)	97	(23.7)
Spouse diagnosed with serious disease (*n = 408)	12	(2.9)
Incapability to express wills (*n = 415)	13	(3.1)
At least one of above psychosocial factors	265	(63.5)
Active addictions (tobacco, alcohol, other drug) (*n = 410)	115	(28.1)
Number of serious co-morbidities (*n = 413)		
0	148	(35.8)
1	180	(43.6)
2 or more	85	(20.6)
**Primary cancer site*(n = 416)***		
Lung	99	(23.8)
Sarcoma (bone or soft tissue)	79	[19]
Urothelial and bladder	57	(13.7)
Pancreas	23	(5.5)
Liver or biliary tract	23	(5.5)
Gastrointestinal	23	(5.5)
Kidney	21	(5.0)
Prostate	20	(4.8)
Breast	19	(4.6)
Endometrium and cervix	13	(3.1)
Ovarian	11	(2.6)
Other (endocrine, dermatologic, unknown primary)	28	(6.7)
Disease deemed incurable(*n = 415)		
at the time of initial diagnosis	231	(55.7)
≤ 6 month after initial diagnosis	45	(10.8)
> 6 month after initial diagnosis	132	(31.8)
Deemed curable	7	(1.7)

*OPM* onco-palliative meetings

Patients were mostly men (*n* = 249/416; 59.9%) with a mean age of 62 years. They presented with some kind of complexity since 63.7% (*n* = 265/416) of patients had at least one factor of social vulnerability, 28.1% (*n* = 115/410) had an active addiction and 64.2% (*n* = 265/413) had at least one serious medical comorbidity.

The main types of cancer were lung cancer (23.8%); bone or soft tissue sarcoma (19%); and urothelial and bladder cancers (13.7%). At the time of the initial diagnosis, 55.7% (*n* = 231/415) of patients were considered to have an incurable disease (locally advanced or metastatic).

### Timing of the first referral to the IOPC program regarding the course of the disease

**Fig. 3:**
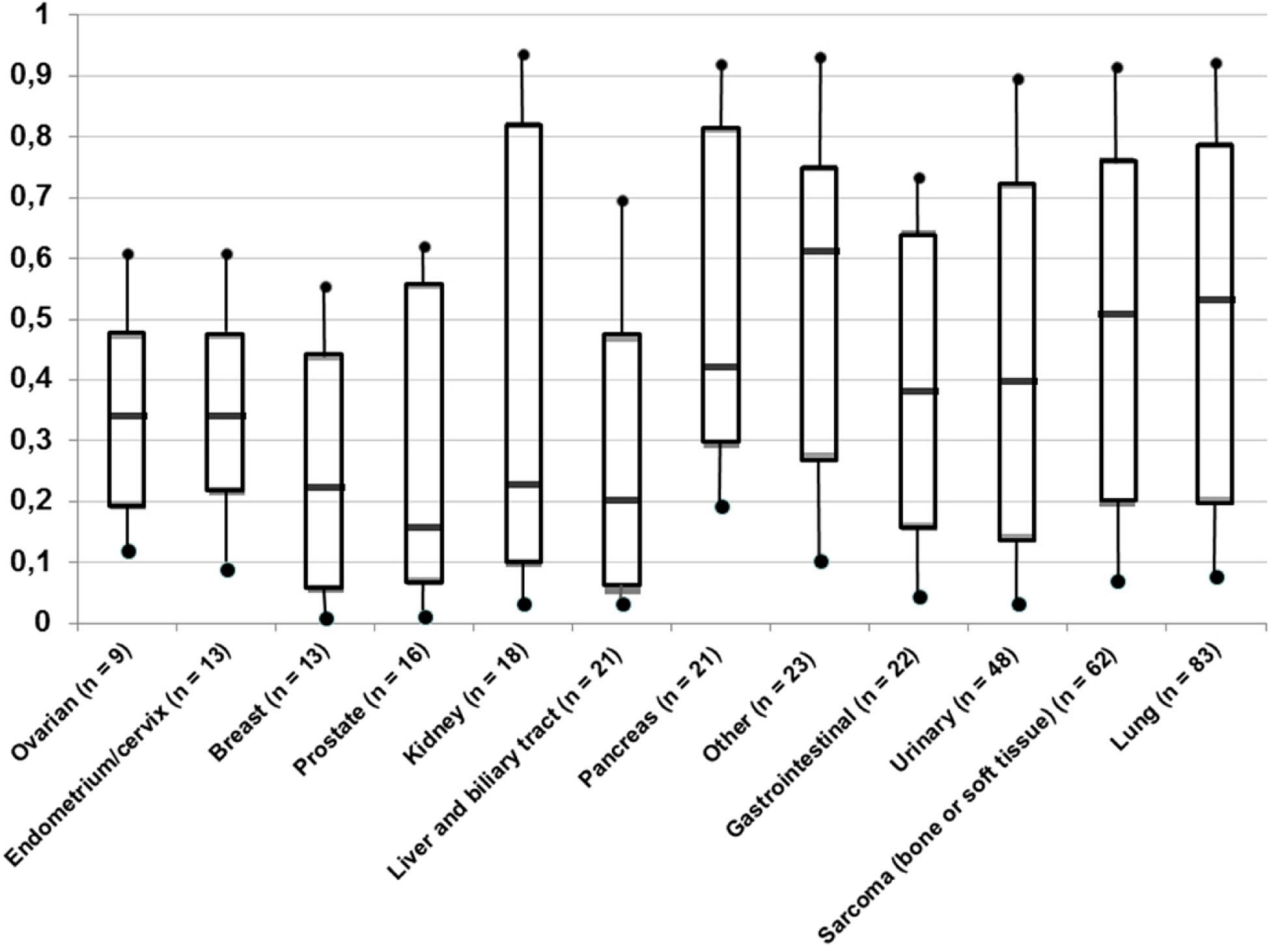
Index of Precocity of first referral to the integrated oncology and palliative care program (IOPC), according to cancer type (0 = late to 1 = early referral)

The characteristics relative to the context of patients’ care are reported in Table 2. At the time of the referral to the IOPC program, 65.2% (*n* = 270/414) of patients had a progressive disease, whereas 26.6% (*n* = 110/414) were at diagnosis and 8.2% (*n* = 34/414) were stable or responded to their last oncologic treatment. Seventy-six percent of patients (*n* = 314/413) were receiving antitumoral treatment or were about to. The majority of patients received ambulatory care (*n* = 256/406; 63.1%). When they could be found in health records, collected prognosis factors showed that 44.3% of patients had a ECOG-PS ≥ 3 (*n* = 176/397), serum albumin levels were < 35 g/l for 54.2% of patients (194/358). A large majority of patients had a CRP > 5 g/l (87.3%; *n* = 308/353). The overall median [1st - 3rd quartile] index of precocity was 0.39 [0.16–0.72]. The medians of each disease-specific index of precocity are represented in Fig. 3. They range from 0.53 [0.20–0.79] (the earliest, for lung cancer) to 0.16 [0.07–0.56] (the latest, for prostate cancer). The median index of precocity was of 0.51 [0.20–0.76] for bone or soft tissue sarcoma, and of 0.40 [0.14–0.72] for urothelial and bladder cancers.

**Table 2:** Context of the first referral to the integrated oncology and palliative care program (IOPC), relatively to the course of disease and project of care

	N	%
(*total number of subjects specified in the case of missing data)	*(or median)*	*(or Q1-Q3)*
Stage of disease(*n = 414)		
At diagnosis	110	26,6
Stability of tumour or positive response to last treatment	34	8,2
Tumour progression	270	65,2
Antitumoral treatment use at the moment of 1st OPM(*n = 413)		
Treatment on course	222	53,8
Treatment to come or 1st line not yet evaluated	92	22,3
No treatment considered	29	7,0
Treatment definitely discontinued	70	16,9
Setting of care at the moment of 1st OPM(*n = 406)		
Inpatient care	150	36,9
Ambulatory / Outpatient clinic	256	63,1
**Individual prognostic factors**		
ECOG-Performance status (PS) (*n = 397)		
≤ 2	221	55,7
≥ 3	176	44,3
Serum albumin level, g/L (*n = 358) median (Q1-Q3)	*34*	(29–38)
≥ 35	164	45,8
28–34	125	34,9
< 28	69	19,3
Serum C-reactive protein level, g/L (*n = 353) median (Q1-Q3)	*40*	*(13–85)*
> 5	308	87,3
≤ 5	*45*	12,7
Serum Lymphocytes level, /mm^3^(*n = 372) median (Q1-Q3)	*1135*	*(800–1603)*
≥ 1500	111	29,8
700–1500	194	52,2
< 700	67	18,0
Serum Lactate Dehydrogenase level, UI/L (*n = 156) median (Q1-Q3)	*441*	*(339–670)*
≥ 400	93	59,6
< 400	63	40,4

*OPM* onco-palliative meetings, *ECOG-Performance status* Eastern Cooperative Oncology Group –Performance status

Note: The prognostic factors collected were measured within 7 days of 1st referral to the IOPC program

ECOG-Performance status [23]:

Grade 0: Fully active, able to carry on all pre-disease performance without restriction

Grade 1: Restricted in physically strenuous activity but ambulatory and able to carry out work of a light or sedentary nature, e.g., light house work, office work

Grade 2: Ambulatory and capable of all self-care but unable to carry out any work activities; up and about more than 50% of waking hours

Grade 3: Capable of only limited self-care; confined to bed or chair more than 50% of waking hours

Grade 4: Completely disabled; cannot carry on any self-care; totally confined to bed or chair

Grade 5: Dead

**Fig. 4:**
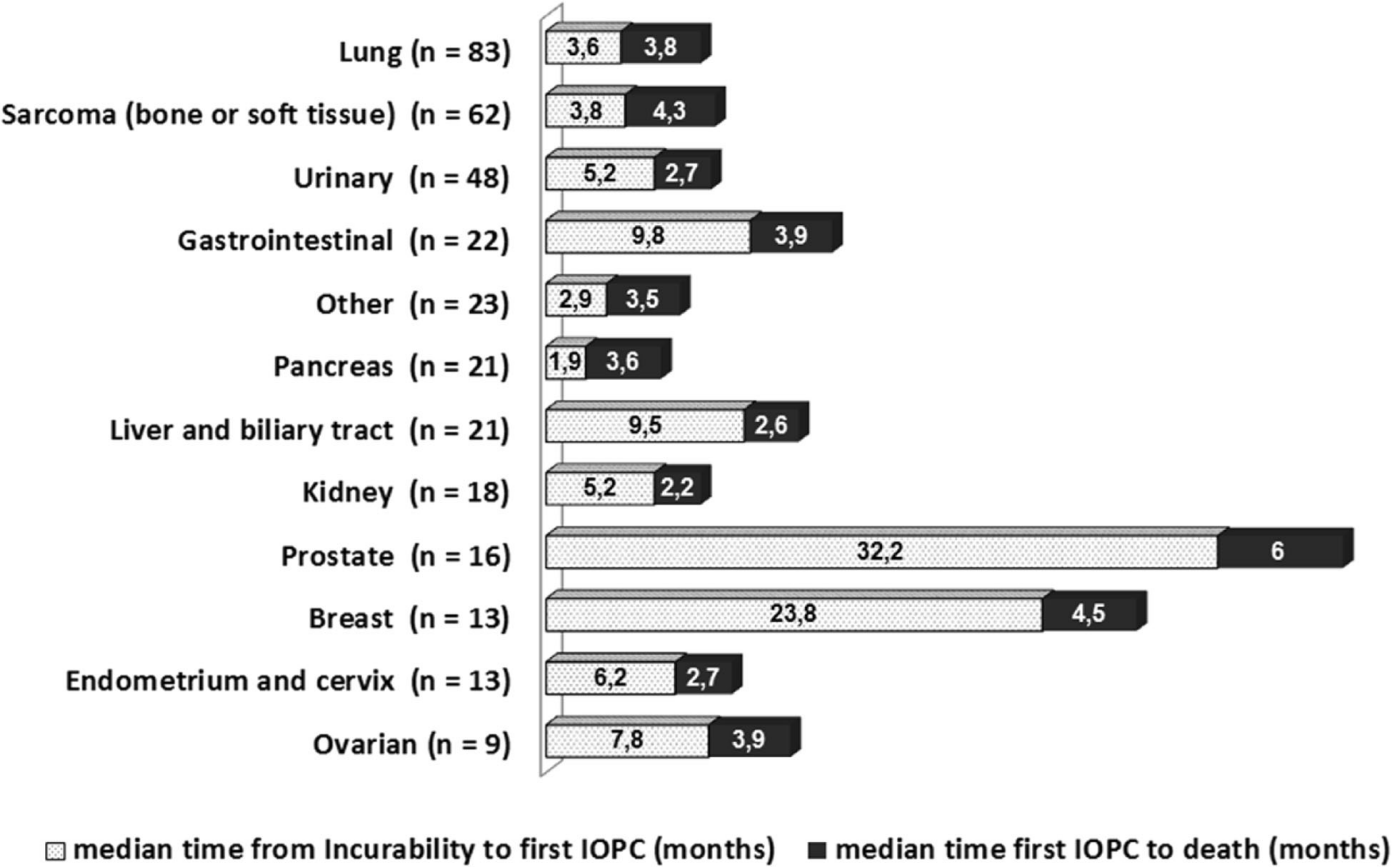
Timing of first referral to the integrated oncology and palliative care program, according to incurability and death

Whereas the disease-specific index of precocity represents the timing of referral to IOPC, relatively to the duration of the progression of metastatic disease, the Fig. 4 represents a more absolute view of this timing: it represents the median time from incurability to 1st referral to the IOPC program stacked with the median length of survival after 1st referral to the IOPC program. These two representations together provide an overall picture of the timing of referral to the IOPC program, by cancer site.

### Trajectories and aggressiveness of care near the end-of-life

Indicators of end-of-life care are reported in Table 3 for the 367 patients who died by the end of follow up. Among them, 44.4% (*n* = 162/365) received one or more new lines of antitumoral treatment after the first referral to the IOPC program. 12.7% (*n* = 42/332) received antitumoral treatment during their last 14 days of life. Half of the patients died at home or in a palliative care unit (50.7%; *n* = 186), whereas 37.1% (*n* = 136) of patients died in an acute care service and 4.6% (*n* = 17) died in the emergency department or in intensive care units.

**Table 3:** Trajectories and indicators of end-of-life aggressiveness of care *(n = 367 decedents)*

	N	%
(*total number of subjects specified in the case of missing data)	*(or median)*	*(or Q1-Q3)*
Length of survival after diagnosis of incurability (months),(*n = 361)	*11,1*	*(5,2 - 22,2)*
Length of survival after first IOPC (months),(*n = 362)	*3.7*	*(1,4 - 7,5)*
Index of Precocity of IOPC(*n = 349)	*0,39*	*(0,16 - 0,72)*
Number of new lines of antitumoral treatment after 1st IOPC(*n = 365)		
0	203	55,6%
1	98	26,8%
≥ 2	64	17,5%
Antitumoral treatment in the last 14 days of life(*n = 332)	42	12,7%
Location of death(*n = 367)		
Acute care hospital	136	37,1%
Emergency or Intensive care unit	17	4,6%
Rehabilitation unit	10	2,7%
Palliative care units	157	42,8%
Home	29	7,9%
Unknown	18	4,9%
Admission to palliative care units within 3 days of death(*n = 141)	18	12,8%

*IOPC* integrated oncology and palliative care program

Note: The Index of precocity (IP) is the ratio of the length of survival after first referral to the IOPC program by the length of survival after diagnosis of incurability. Its values lie between 0 (referral to the IOPC program occurs late, close before death) and 1 (referral to the IOPC program occurs early after the diagnosis of incurability)

## Discussion

In this study, we analyzed the profiles of 416 cancer patients at the moment of the first referral to the IOPC program (including either the first report to OPM, or the first referral to the PCT). Most of the patients received ambulatory care, their cancers were considered to be in progression and a large majority was still receiving antitumoral treatment. Individual prognosis factors collected showed aggressive disease. Half of the patients were referred to the IOPC program within 3.7 months before death (median survival after first referral to the IOPC program). Disease-specific index of precocity showed a great variability of the timing of referral according to cancer localisation, which reflects different natural histories of diseases.

Indicators of trajectory and aggressiveness of care at the end-of-life have been collected to describe patients’ trajectory of care after referral to the IOPC program. Our results showed limited use of chemotherapy near the end-of-life and a relatively high rate of death in palliative care units, which is close to the standards proposed by Earle et al [30]: 12.7% of patients received chemotherapy in the last 14 days, 50.9% of patients died either at home or in palliative care units and admission to palliative care units within 3 days before death occurs for 12.8% of patients. However, our results on the location of death (44.7% of patients died in hospital, emergency room or intensive care units, or rehabilitation unit) are to be interpreted in the French healthcare system in which home care is underdeveloped, as shown by the national mortality data (18.9% of cancer death occurring at home) [31]. This proportion is even lower in our population, as it has been described in similar population from university hospitals [6].

As a consequence of both retrospective design and collection of data by chart review, missing data for some characteristics can skew the estimate of their frequencies. As factors of vulnerability are less likely to be traced in patients’ record when they are absent, we can assume that our results are overestimated. For prognostic factors, it is more difficult to interpret the sense of bias as the lab test order for prognosis investigation was still not a systematic practice in our setting at the time of the study, which remains an issue.

The main issue of palliative care integration in oncology is the question of the timing of this integration. In this study, this timing has to be interpreted in the context of our hospital where the population is not representative of the general oncologic patient population. Bone or soft tissue sarcoma are over-represented (our hospital being a centre of reference for those tumours), whereas breast cancers are under-represented (low number of patients followed for breast cancer and mostly after request for second opinions). Moreover, our population is composed of critically ill patients with half of the patients being diagnosed at an advanced stage of their disease. Our practice of referral to the IOPC program differs from the early palliative care model evaluated by Temel et al [13], where all patients are referred to the PCT within 8 weeks of the diagnosis of metastatic lung cancer, have a PS ≤ 2 and receive antitumoral treatment. Despite this difference of model, the median time between first referral to the IOPC program and death was 3.7 months, which is earlier than the one observed in practices contemporaneous to our study [6, 32, 33].,

In their effort to establish consensual referral criteria, Hui et al. defined two time based criteria: “within 3 months of diagnosis of advanced or incurable cancer for patients with median survival of 1 year or less” and at “diagnosis of advanced cancer with progressive disease despite second-line systemic therapy (incurable)” [11]. The heterogeneity of natural histories of different tumour types leads other authors to propose disease-specific timing of palliative care integration [34]. For example, in the case of metastatic breast or prostate cancer, the rather chronic course of the disease brings to question the diagnosis of metastasis as the right moment for integration. In the study of Zimmermann et al [14], comparing systematic consultation and follow-up by PCT versus standard care, eligible patients were defined as having a stage IV cancer except for breast and prostate cancer for which refractory to hormonal therapy was an additional criterion. Moreover, the constant therapeutic advances should also be taken into account in the evolution of the disease. With the development of targeted therapies and immunotherapy [20, 35–37],, this question will probably raise for other tumour localisations such as lung cancer or melanoma.

To take these evolutions into account, we propose the index of precocity which describes the moment of palliative care integration relatively to the course of the disease. As an example, in our study, integration was early in the course of the disease for patients with lung cancer, as recommended since 2012 [2], or sarcoma, which can be explain in our experience by the high burden of physical or psychosocial symptoms occurring early in the trajectory. The index of precocity was the lowest for prostate cancer (0.16 [0.07–0.56]), as expected by the long efficacy of hormonotherapy in metastatic phase, whereas the median length of survival after 1st referral to the IOPC program was the longest one in absolute value for this tumor site (6 months).

## Conclusion

The model of palliative care integration in oncology should remain close to the experimental early palliative care model which has been proved to increase the quality of life of patients. However, the optimized use of palliative care resources is essential to make them accessible to all patients who will benefit from them. Patients should be referred to palliative care at the right time for the right reasons. In the early phase of long lasting incurable disease, patients with no uncontrolled symptoms and no psychosocial needs have no a priori reason to benefit from palliative care. In the model we described, the shared-discussion process that took place in OPM worked as a screening tool to identify patients who will benefit from a palliative care program. In the screening process both time-based and needs-based criteria are taken into account. To evaluate its feasibility and adaptability in other setting, this model should be experimented in other French hospitals. In a perspective of practice analyses in multicentre setting, the index of precocity could be an interesting tool to describe actual integration of palliative care, adjusting for the duration of incurability, and to highlight any inter-centre differences in the implementation of the same model. It will therefore be interesting to prospectively examine whether this index is predictive of the aggressiveness of care near the end-of-life.

## Data Availability

No specific data was collected for the study, except data routinely collected and recorded in electronic patient files. Between 2011 and 2013 (period of the retrospective monocentre study), patients’ verbal consent to record clinical data in hospital information system was systematically obtained from inpatients and patients visiting outpatient clinic, along with its use for public interest research purpose. We therefore did not need any permission to access the data analyzed and reported in this study. However, at the study time, the transmission of data to a third party was not included in the information form. Nowadays, current regulations in France and Europe do not allow the transmission of personal data without the data subjects having been informed of the transmission to a third party. For this reason, the dataset generated and analysed for the current study can only be made available upon reasonable request to the corresponding author and with restrictions for full anonymysation.

## Abbreviations

ECOG-PS: Eastern Cooperative Oncology Group - Performance status
IOPC program: Integrated Onco-Palliative Care program
IP: Index of precocity
OPM: Onco-palliative meeting
PC: Palliative care
PCT: Palliative care consultation teams
